# Supplementary material for: Occurrence and Multidrug Resistance of Campylobacter in Chicken Meat from Different Production Systems
Source: Foods. 2022 Jun 21;11(13):1827. doi: 10.3390/foods11131827 (PMC9265442; doi:10.3390/foods11131827)
Supplement: Supplementary file 1 [file foods-11-01827-s001.zip › Santos-Ferreira_Suplementary Figure S2_Subm.pdf]

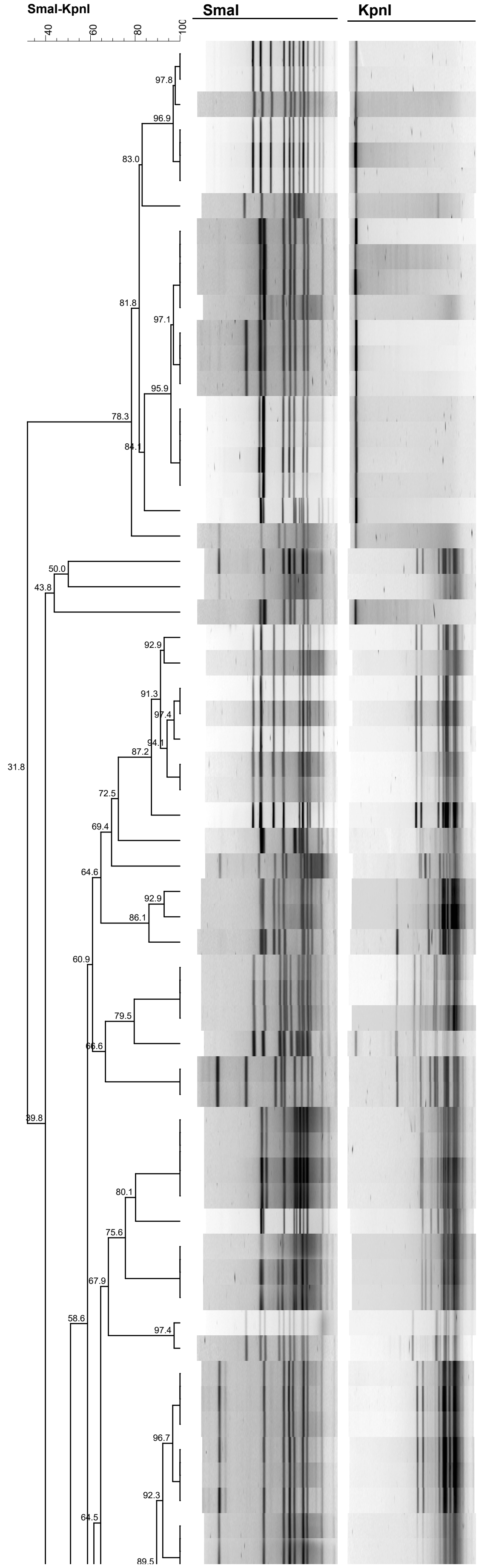

isolate code	Sample	Method	AMP	AMC	CN	CIP	E	TE	IMP	MEM	NA
GC2/10	GC2	Enumeration	R	S	S	R	R	R	S	S	R
GC2/17	GC2	Enumeration	R	S	S	R	R	R	S	S	R
GC5/10	GC5	Detection	R	I	S	R	R	R	S	S	R
GC2/3	GC2	Enumeration	R	S	S	R	R	R	S	S	R
GC2/8	GC2	Enumeration	R	S	S	R	R	R	S	S	R
GC2/9	GC2	Enumeration	R	S	S	R	R	R	S	S	R
GC7/2	GC7	Detection	R	I	S	R	S	R	S	S	R
BY5/15	BY5	Detection	R	S	S	R	S	R	S	S	R
GC3/13	GC3	Detection	R	S	S	R	S	R	S	S	R
GC3/14	GC3	Detection	R	S	S	R	S	S	S	S	R
GC4/1	GC4	Enumeration	R	R	S	R	S	R	S	S	R
BY5/13	BY5	Detection	R	S	S	R	S	R	S	S	R
BY5/14	BY5	Detection	R	S	S	R	S	R	S	S	R
BY5/16	BY5	Detection	R	S	S	R	S	R	S	S	R
GC4/2	GC4	Enumeration	R	S	S	R	S	R	S	S	R
GC4/4	GC4	Enumeration	R	S	S	R	S	R	S	S	R
GC4/8	GC4	Detection	R	I	S	R	S	R	S	S	R
GC4/10	GC4	Detection	R	S	S	R	S	R	S	S	R
BY9/9	BY9	Detection	S	S	S	R	S	R	S	S	R
GC5/4	GC5	Enumeration	R	I	S	R	S	R	S	S	R
RT1/13	RT1	Detection	I	S	S	R	S	R	S	S	R
RT1/18	RT1	Detection	I	S	S	R	S	R	S	S	R
GC3/19	GC3	Detection	R	S	S	R	S	S	S	S	R
BY3E7	BY3	Detection	R	S	S	R	S	R	S	S	R
BY2E4	BY2	Detection	R	S	S	R	S	R	S	S	R
BY3E1	BY3	Detection	R	S	S	R	S	R	S	S	R
BY2E1	BY2	Detection	R	S	S	R	S	R	S	S	R
BY3E5	BY3	Detection	R	S	S	R	S	R	S	S	R
BY2E5	BY2	Detection	R	S	S	R	S	R	S	S	R
BY2E6	BY2	Detection	R	S	S	R	S	R	S	S	R
BY3E6	BY3	Detection	R	S	S	R	S	R	S	S	R
C2D2	C2	Enumeration	R	I	S	R	S	R	S	S	R
RT9/6	RT9	Enumeration	R	S	S	R	S	R	S	S	R
RT5/15	RT5	Detection	R	S	S	R	R	R	S	S	R
RT5/16	RT5	Detection	R	S	S	R	R	R	S	S	R
GC5/2	GC5	Enumeration	R	S	S	R	R	R	S	S	R
RT5/1	RT5	Enumeration	R	I	S	R	S	R	S	S	R
RT5/2	RT5	Enumeration	R	I	S	R	S	R	S	S	R
RT5/3	RT5	Enumeration	R	S	S	R	S	R	S	S	R
RT3/7	RT3	Enumeration	R	S	S	R	S	R	S	S	R
GC3/17	GC3	Detection	R	S	S	R	R	R	S	S	R
GC3/18	GC3	Detection	R	S	S	R	R	R	S	S	R
RT4/1	RT4	Enumeration	R	S	S	R	R	R	S	S	R
RT4/6	RT4	Detection	R	I	S	R	R	R	S	S	R
RT4/7	RT4	Detection	R	S	S	R	R	R	S	S	R
RT4/8	RT4	Detection	R	S	S	R	R	R	S	S	R
GC2/4	GC2	Enumeration	R	S	S	R	R	R	S	S	R
RT8/7	RT8	Enumeration	S	S	S	R	S	R	S	S	R
RT8/9	RT8	Detection	S	S	S	R	S	R	S	S	R
RT8/13	RT8	Detection	S	S	S	R	S	R	S	S	R
BY9/8	BY9	Detection	R	S	S	R	R	R	S	S	R
BY9/10	BY9	Detection	R	S	S	R	R	R	S	S	R
GC8/2	GC8	Detection	R	S	S	R	S	R	S	S	R
GC8/3	GC8	Detection	R	I	S	R	S	R	S	S	R
GC8/4	GC8	Detection	R	S	S	R	S	R	S	S	R
BY10/8	BY10	Detection	R	S	S	R	S	R	S	S	R
BY10/9	BY10	Detection	R	S	S	R	S	R	S	S	R
BY10/10	BY10	Detection	R	S	S	R	S	R	S	S	R
RT1/15	RT1	Detection	R	S	S	R	S	R	S	S	R
RT1/16	RT1	Detection	I	S	S	R	S	R	S	S	R

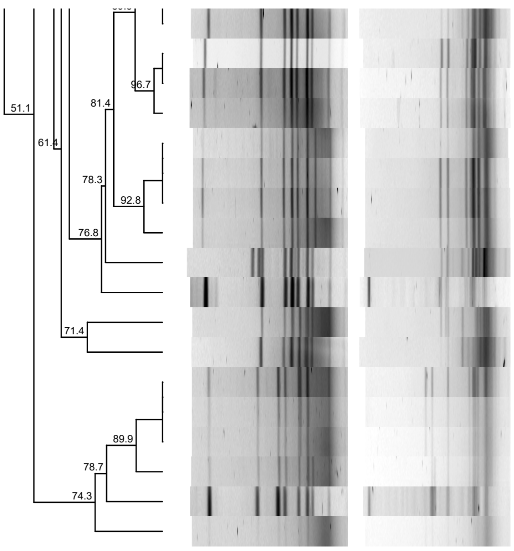

RT1/17	RT1	Detection	I	S	S	R	S	R	S	S	R
GC6/10	GC6	Detection	S	S	S	R	S	R	S	S	R
GC6/7	GC6	Detection	I	S	S	R	S	R	S	S	R
GC6/9	GC6	Detection	I	S	S	R	S	R	S	S	R
BY11/2	BY11	Detection	S	S	S	R	S	R	S	S	R
BY11/3	BY11	Detection	S	S	S	R	S	R	S	S	R
BY11/4	BY11	Detection	S	S	S	R	S	R	S	S	R
BY11/5	BY11	Detection	S	S	S	R	S	R	S	S	R
RT7/4	RT7	Enumeration	R	I	S	R	R	R	S	S	R
GC6/8	GC6	Detection	S	S	S	R	S	R	S	S	R
RT1/10	RT1	Enumeration	R	S	S	R	R	R	S	S	R
RT1/8	RT1	Enumeration	R	S	S	R	R	R	S	S	R
RT11/2	RT11	Enumeration	R	I	S	R	S	R	S	S	R
RT11/4	RT11	Enumeration	R	I	S	R	S	R	S	S	R
RT11/6	RT11	Enumeration	R	I	S	R	S	R	S	S	R
RT11/7	RT11	Enumeration	R	S	S	R	S	R	S	S	R
GC5/9	GC5	Detection	R	I	S	R	S	R	S	S	R
RT11/5	RT11	Enumeration	R	I	S	R	S	R	S	S	R
