# Supplementary material for: Occurrence and Multidrug Resistance of Campylobacter in Chicken Meat from Different Production Systems
Source: Foods. 2022 Jun 21;11(13):1827. doi: 10.3390/foods11131827 (PMC9265442; doi:10.3390/foods11131827)
Supplement: Supplementary file 1 [file foods-11-01827-s001.zip › Santos-Ferreira_Supplementary Table 1_subm.pdf]

Supplementary Table S1. Distribution of antibiotic susceptibility of *Campylobacter* spp. isolated from conventionally reared, free-range and backyard chicken samples.

Production System	Suscep-tibility[a]	Antibiotic[b] No. of isolates (%)								
		AMP	AMC	CN	CIP	E	TE	IMP	MEM	NA
Campylobacter coli										
Free Range	R	25 (86.2%)	1 (3.4%)	0	29 (100%)	10 (34.5%)	27 (93.1%)	0	0	29 (100%)
	I	2 (6.9%)	7 (24.1%)	0	0	0	0	0	0	0
	S	2 (6.9%)	21 (72.4%)	29 (100%)	0	19 (65.5%)	2 (6.9%)	29 (100%)	29 (100%)	0
Conventionally reared	R	20 (74.1%)	0	0	27 (100%)	89(33.3%)	27 (100%)	0	0	27 (100%)
	I	4 (14.8%)	8 (29.6%)	0	0	0	0	0	0	0
	S	3 (11.1%)	19 (70.4%)	27 (100%)	0	18 (66.7%)	0	27 (100%)	27 (100%)	0
Backyard	R	17 (77.3%)	0	0	22 (100%)	2 (9.1%)	22 (100%)	0	0	22 (100%)
	I	0	0	0	0	0	0	0	0	0
	S	5 (22.7%)	22 (100%)	22 (100%)	0	20 (90.9%)	0	22 (100%)	22 (100%)	0
TOTAL	R	62 (79.5%)	1 (1.3%)	0	78 (100%)	21 (26.9%)	76 (97.4%)	0	0	78 (100%)
	I	6 (7.7%)	15 (19.2%)	0	0	0	0	0	0	0
	S	10 (12.8%)	62 (79.5%)	78 (100%)	0	57 (73.1%)	2 (2.6%)	78 (100%)	78 (100%)	0
Campylobacter jejuni										
Free Range	R	15 (88.2%)	0	0	15 (88.2%)	1 (5.9%)	15 (88.2%)	0	0	15 (88.2%)
	I	1 (5.9%)	0	0	0	0	0	0	0	0
	S	1 (5.9%)	17 (100$)	17 (100%)	2 (11.8%)	16 (94.1%)	2 (11.8%)	17 (100%)	17 (100%)	2 (11.8%)
Conventionally reared	R	23 (100%)	0	0	23 (100%)	4 (17.4%)	23 (100%)	0	0	23 (100%)
	I	0	0	0	0	0	0	0	0	0
	S	0	23 (100%)	23 (100%)	0	19 (82.6%)	0	23 (100%)	23 (100%)	0
Backyard	R	10 (83.3%)	0	0	12 (100%)	0	10 (83.3%)	0	0	12 (100%)
	I	0	0	0	0	0	0	0	0	0
	S	2 (16.7%)	12 (100%)	12 (100%)	0	12 (100%)	2 (16.7%)	12 (100%)	12 (100%)	0
TOTAL	R	48 (92.3%)	0	0	50 (96.2%)	5 (9.6%)	48 (92.3%)	0	0	50 (96.2%)
	I	1 (1.9%)	0	0	0	0	0	0	0	0
	S	3 (5.8%)	52 (100)	52 (100)	2 (3.8%)	47 (90.4%)	4 (7.7%)	52 (100%)	52 (100%)	2 (3.8%)

[a] S – Susceptible, I – Intermediate, R – Resistant

[b]AMP – ampicillin (10 µg), AMC - amoxicillin/clavulanic acid (30 µg), GEN – gentamicin (10 µg), CIP – ciprofloxacin (5 µg), NAL - nalidixic acid (30 µg), ERY-erythromycin (15 µg), TET – tetracycline (30 µg), IPM – imipenem (10 µg), and MEM – meropenem (10 µg).
