# Supplementary figures and images for: Occurrence and Multidrug Resistance of Campylobacter in Chicken Meat from Different Production Systems
Source: Foods. 2022 Jun 21;11(13):1827. doi: 10.3390/foods11131827 (PMC9265442; doi:10.3390/foods11131827)

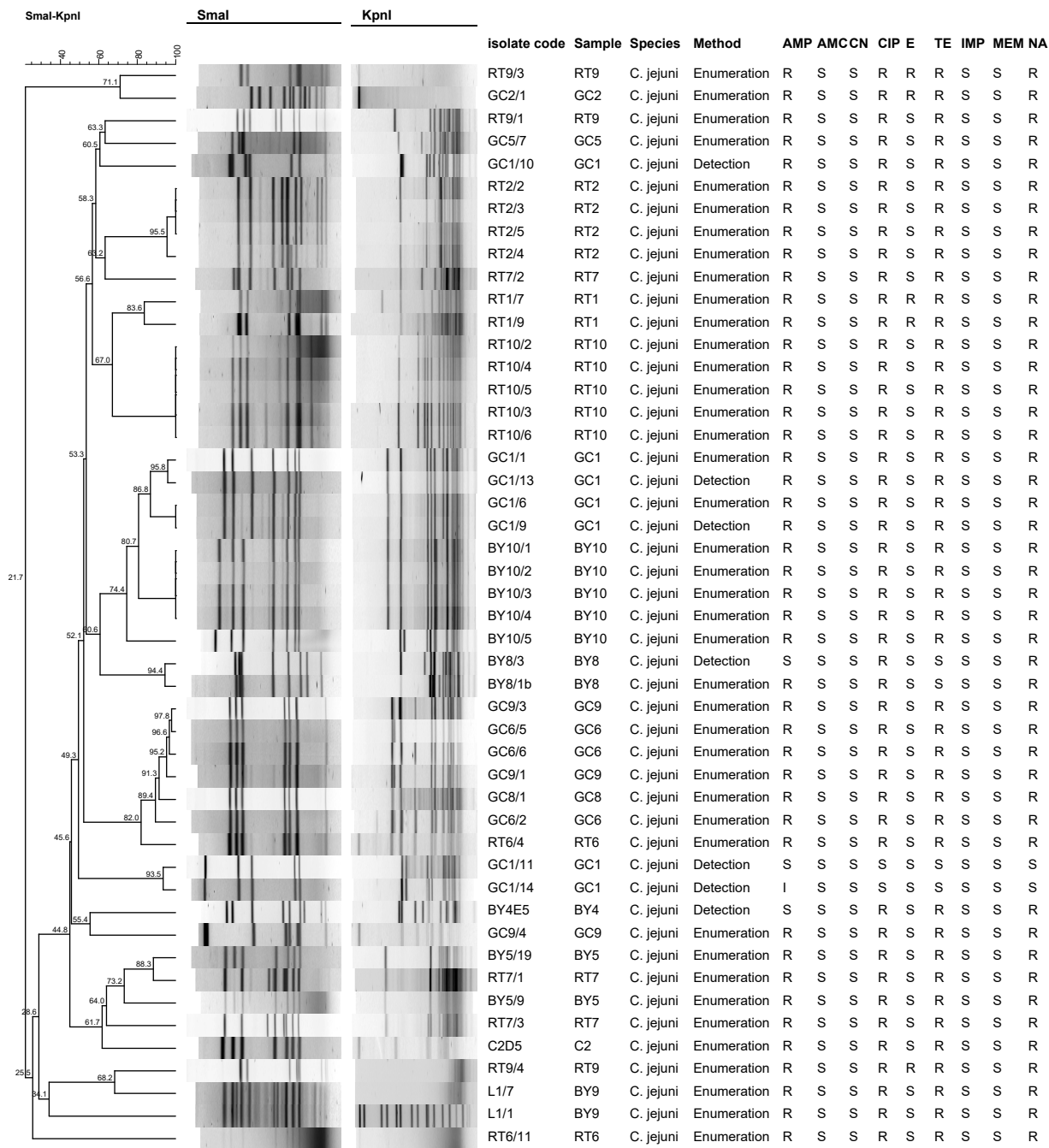

Supplement: Supplementary file 1 [file foods-11-01827-s001.zip › Santos-Ferreira_Supplementary Figure S1_Subm.pdf]
